# Sodium Valproate-Induced Chromatin Remodeling

**DOI:** 10.3389/fcell.2021.645518

**Published:** 2021-04-20

**Authors:** Maria Luiza S. Mello

**Affiliations:** Department of Structural and Functional Biology, University of Campinas (Unicamp), Campinas, Brazil

**Keywords:** chromatin, sodium valproate, epigenetics, histones, DNA, image analysis, FTIR

## Abstract

Valproic acid/sodium valproate (VPA), a drug originally prescribed as an anticonvulsant, has been widely reported to act on epigenetic marks by inducing histone acetylation, affecting the DNA and histone methylation status, and altering the expression of transcription factors, thus leading to modulation of gene expression. All these epigenetic changes have been associated with chromatin remodeling effects. The present minireview briefly reports the main effects of VPA on chromatin and image analysis and Fourier transform infrared (FTIR) microspectroscopy in association with molecular biology methodological approaches to investigate the VPA-induced changes in chromatin structure and at the higher-order supraorganizational level.

## Introduction

Chromatin, which in eukaryotic cells is a complex structure containing DNA, histones, non-histone proteins and RNA, has a dynamic organization essential for its normal physiological performance. It is well known that changes in gene expression may affect chromatin structure at the molecular level. Chromatin remodeling also affects chromatin at the superstructural level. Gene expression is modulated temporally and spatially by a series of epigenetic marks that affect specifically DNA and histones. Removal or association of epigenetic marks from/to chromatin components may dramatically change gene expression and chromatin structure. In this context, drugs that inhibit histone deacetylases (HDACi), facilitate the access of acetyl groups to histones or interfere with the activity of methyltransferases that control the methylation status of DNA and histones, have a role affecting gene expression that is generally accompanied by chromatin remodeling. One example of such a drug is valproic acid (VPA), which was originally prescribed for the treatment of seizure disorders and was subsequently revealed to be a potent epigenetic agent.

Improvements in assessing changes in chromatin architecture associated with the modulation of epigenetic marks have been developed to better understand alterations in chromatin functionality. Thus, examination of the known of VPA action, including those concerned with chromatin remodeling, may be instrumental in making decisions concerning the practical use of this drug.

In this minireview, the main effects of VPA on chromatin epigenetic marks are described, and image analysis and infrared spectroscopic methodological approaches to demonstrate VPA-induced changes in chromatin structure and higher-order superstructure are briefly reported.

## Valproic Acid/Sodium Valproate Effects on Chromatin Components

Valproic acid is a short-chain fatty acid. The effects of VPA associated with its prescription for the treatment of seizure disorders, including epilepsy, are primarily mediated by its activities as an inhibitor of GABA transaminase and blocker of voltage-gated sodium channels and T-type calcium channels (Chateauvieux et al., 2010). Drug pharmacological formulations require the association of VPA with its sodium salt (sodium valproate), to obtain a solid compound convenient for storage and human oral administration (Petrusevski et al., 2008).

The first demonstration that VPA could affect epigenetic markers and chromatin structure came with the demonstration that it inhibited class I histone deacetylases (HDACs), which favor histone acetylation, especially at the lysine 9 residue of histone H3 and the lysine 8 residue of histone H4 (Göttlicher et al., 2001; Phiel et al., 2001; Eyal et al., 2004). With VPA-induced histone H4 hyperacetylation, transcription from diverse promoters can be activated, cell cycle arrested, and apoptosis intrinsic and extrinsic pathways elicited. In HeLa human cervical carcinoma cells, treatment with 3.0 mM VPA for 24 h, which induces histone H4 hyperacetylation, led to gene deregulation, with upregulation of more than twofold of 1,074 genes (including genes related to the cell cycle, cell signaling, pyruvate dehydrogenase kinase 4 and ATPase class V) and downregulation of 551 genes (including genes related to importin β, Fas apoptotic inhibitory molecule, and cyclin B1) (Dejligbjerg et al., 2008). In VPA-treated rat neurons, increased acetylation of histones H3 and H4 was detected only in the promoters of the upregulated genes, and was found to affect 726 genes, including genes involved in epileptogenesis (Fukuchi et al., 2009).

The importance of HDAC inhibitors, including suberoylanilide hydroxamic acid (SAHA) and trichostatin A (TSA), in neuronal differentiation and neuroprotection, has been recently reviewed by Shukla and Tekwani (2020). When comparing the effects of VPA, SAHA, and TSA on neurogenesis, a higher number of differentially expressed genes and a more potent dysregulation of stem cell differentiation have been found to result from the VPA action (Meganathan et al., 2015). VPA-induced upregulation of axonogenesis and ventral forebrain-associated genes and repression of neural tube and dorsal forebrain transcripts occur *via* miR-378 microRNA (Meganathan et al., 2015). Although HDAC inhibitors may play a role in the pathogenesis of neurodegenerative diseases, there are indications that SAHA and VPA may act as potential neurotrophins (Shukla and Tekwani, 2020).

Valproic acid has also been found to induce chromatin decondensation that lasts longer than the time assigned to promote histone acetylation. This finding suggested that VPA could affect the methylation status of DNA and histones, which was confirmed in several cell types, including tumor cells (Detich et al., 2003; Milutinovic et al., 2007; Marinova et al., 2011; Palsamy et al., 2014; Rocha et al., 2019). The VPA-promoted demethylation of DNA, which leads to the conversion of 5-methylcytosine (5mC) to cytosine (C), involves a complex process flowing through an active or a passive pathway, depending on the cell type. In MCF-7 human breast tumor cells, for instance, VPA-induced DNA demethylation occurs through a passive pathway (Marchion et al., 2005). In human lens epithelial cells and HeLa cells, VPA acts predominantly within the active DNA demethylation pathway, through the action of enzymes of the *ten-eleven translocation* (TET) protein family and independent of cell replication, although a passive pathway promoting the suppression of DNA methyltransferase (DNMT) activity may also be involved (Palsamy et al., 2014; Rocha et al., 2019). Although reversible, DNA methylation changes are more stable than histone acetylation alterations, and may lead to long-term epigenetic reprogramming (Milutinovic et al., 2007). In response to VPA, a dynamic interplay has also been verified between the acetylation of histone tails and changes in DNA methylation, including decreased methylation of tumor suppressor genes (Marchion et al., 2005; Milutinovic et al., 2007; Dong et al., 2010; Gu et al., 2012). These findings suggested that VPA may have therapeutic potential due to its antitumor effects especially when administered in synergistic combination with other agents (Braiteh et al., 2008; Mohammed et al., 2011; Booth et al., 2017; Heers et al., 2018; Zhang et al., 2019).

Methylation and demethylation of histones are other events modulated by VPA. In histone H3 of HEK 293 human embryonic kidney cells, hypermethylation of lysine 4, conferring hypomethylation of lysine 27, occurred simultaneously with HDAC inhibition promoted by VPA (Ganai et al., 2015). Global changes in the abundance of di- and trimethylated lysine 4 and of mono- and dimethylated lysine 9 of histone H3 (H3K4me2, H3K4me3, H3K9me, and H3K9me2, respectively) are present in several tumor types, giving support to the hypothesis that overall histone modifications may represent potential markers of cancer prognosis (Santos-Rosa et al., 2002; Ellinger et al., 2010; Fang et al., 2014; Beyer et al., 2017). In particular, H3K4me2 is associated with a small subset of genes that become prepared for rapid activation in response to stimulus (Russ et al., 2014).

H3K4me3, a marker of the exclusive active state of gene expression (Santos-Rosa et al., 2002), increased significantly in HeLa cells cultivated in the presence of 0.5 and 2 mM VPA, as assessed by immunofluorescence signals and protein abundance (Rocha and Mello, 2020). In addition, immunofluorescence signals of H3K4me2 at the nuclear periphery became intensified in HeLa cells under VPA treatment (Rocha and Mello, 2020). Persistent demethylation of histone H3 lysine 4 at this nuclear region in other cell types has been associated with gene transcriptional activation and is implicated in the generation of a state of transcriptional memory that could last for several days (Gialitakis et al., 2010; Light and Brickner, 2013; Fiserova et al., 2017).

Increased levels of H3K9me concomitant with decreased levels of H3K9me2 may be consistent with the intensification of global gene expression induced by VPA (Rocha and Mello, 2020). In different cell types, H3K9 monomethylation is associated with gene activation, particularly within coding regions (Kouzarides, 2007), whereas H3K9 dimethylation signals are higher in silent genes and formation of heterochromatin (Barski et al., 2007).

Knowledge of the potential effects of VPA continues to be expanded. When HepG2 cells, which are used as an *in vitro* model of diabetes, were exposed to a high-glucose regimen and cultivated in the presence of VPA, RNA-seq assays revealed attenuation of the hyperglycemia-induced activation of complement and coagulation cascade genes (*MASP2, C3*) due to the altered expression of transcription factors (*Hnf-4*α, *Fxr*) (Felisbino et al., 2021). VPA has thus been hypothesized to attenuate some of the deleterious pathways activated by hyperglycemia. Other effects not directly affecting epigenetic markers and unknown until recently, have been proposed after the analysis of mixtures of VPA and DNA and VPA and histones revealed molecular interactions between VPA and chromatin components *in vitro* (Sargolzaei et al., 2017; Vidal and Mello, 2020).

## VPA and Chromatin Remodeling

The fact that chromatin structure is affected by VPA is demonstrated by increased sensitivity of DNA to nucleases and increased association of DNA with intercalating agents (Marchion et al., 2005). The VPA-induced depletion of structural maintenance of chromosome (SMC) proteins 1–5, SMC-associated proteins, DNMT-1 and the heterochromatin protein HP1, resulted in chromatin remodeling and promoted the access of DNA-damaging agents to their target sites (Marchion et al., 2005).

Chromatin decondensation resulting from VPA exposure, when affected at the level of the higher-order hierarchical packing state, often accompanies a decreased HDAC activity and a general increase in histone acetylation and global DNA demethylation. This chromatin decondensation effect can be probed using computer-assisted image analysis procedures, especially when they are validated by immunoassays that identify specific epigenetic targets (Felisbino et al., 2011, 2014; Vidal et al., 2014b; Veronezi et al., 2017). In non-transformed NIH 3T3 cells, these changes have been found to affect not only euchromatin but also constitutive heterochromatin bodies (chromocenters) (Felisbino et al., 2014). Conversely, in other cell systems, such as that of the insect *Triatoma infestans*, only a few cells exhibited heterochromatin decondensation but no histone acetylation in the presence of VPA (Bassani et al., 2021). In this model, such a finding suggests a possible effect of VPA on structural proteins other than histones, which are important for the establishment of the chromatin architecture (Marchion et al., 2005; Kortenhorst et al., 2009). The fact that not all cells within a heterogeneous cell population equally experience chromatin decondensation and response to mechanical stimuli upon treatment with an HDAC inhibitor (for example, TSA) has been revealed in vertebrate cell systems using Fourier transform infrared (FTIR) microspectroscopic imaging (Morrish et al., 2019).

For investigations concerned with molecular changes that affect chromatin structure, FTIR microspectroscopy has been revealed to be a useful analytical approach when global DNA demethylation is induced by VPA treatment (Veronezi et al., 2017). Additionally, when isolated DNA and histones H1 and H3 macromolecules were mixed with VPA, changes in FTIR spectral signatures were used to detect direct interactions between this drug and the chromatin components resulting in conformational alterations in these macromolecules (Vidal and Mello, 2020).

### Image Analysis of Chromatin Suprastructural Changes Under VPA Treatment

**FIGURE 1:**
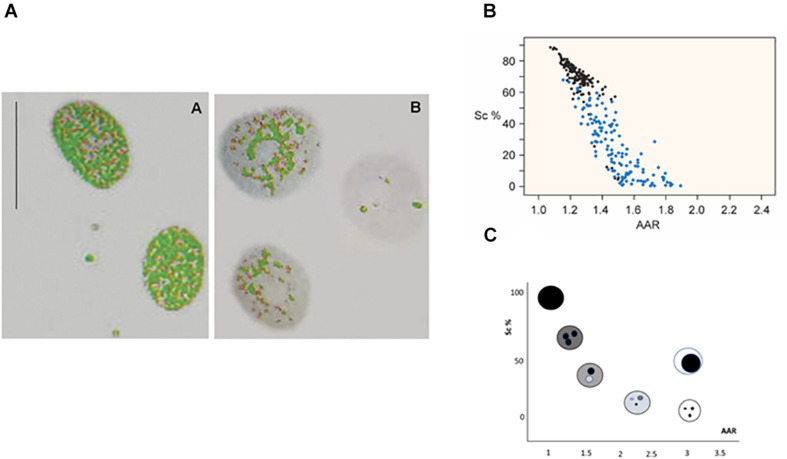
Image analysis of VPA-treated chromatin. Images of VPA-treated Feulgen-stained HeLa cell nuclei show the decreased areas covered by condensed chromatin in gray color **(A)** [reprinted from Felisbino et al. (2011)—PLoS ONE 6: e29144]. A scatter diagram representing the Sc% vs. AAR relationship for Feulgen-stained HeLa cell nuclei shows nuclei treated with 1.0 mM VPA for 2 h (blue dots) compared with nuclei from the respective untreated control (black dots) **(B)** [reprinted from Felisbino et al. (2011)—PloS ONE 6: e29144]. Each dot in the plot represents one nucleus with a specific phenotype as shown in the theoretical model **(C)** [modified from Vidal et al. (2014a)].

Among the various image analysis approaches that permit detecting changes in higher-order chromatin organization, staining of cell nuclei with the DNA-specific Feulgen reaction (Mello and Vidal, 2017) followed by a computer-assisted process of image acquisition, segmentation and featuring, results in the conversion of magenta-colored images into grayscale images that are subsequently converted into false-colored images useful for the evaluation of parameters of interest (Vidal et al., 2014a). By preselecting an absorbance cutoff point in association with a certain higher-order chromatin packing state and applying it to nuclear images in which absorbance values exceed this threshold, a false color such as green, for example, may be automatically attributed to the resulting image. Chromatin decondensation, like that promoted by VPA on histone acetylation and/or DNA demethylation, is revealed microscopically in specific nuclear areas in which the green false color is no longer evident (Figure 1A).

Mathematically, such a change in chromatin architecture can be demonstrated in stained preparations by plotting a scatter diagram containing the distribution of points that corresponds to many nuclear phenotypes of the cell sample at a time (Figures 1B,C). The whole methodological procedure is detailed elsewhere (Vidal et al., 2014a). Briefly, each nuclear phenotype is defined in terms of matching its relative area occupied by condensed chromatin (Sc%) vs. a dimensionless parameter that identifies how many times the average absorbance of the condensed chromatin exceeds the average absorbance of the entire nucleus (AAR) (Vidal, 1984; Vidal et al., 2014a). Consequently, an altered distribution of points in the scatter diagram demonstrates the usefulness of this methodology for studies on chromatin remodeling (Figures 1B,C).

Recently, a proposal was raised to study VPA-induced imaging changes in the nuclear morphology of astrocyte cells in a temporal context, a process that was named 4D morphometry (Kalinin et al., 2021). The authors reported results that reflected chromatin reorganization not only spatially but also temporally, which allowed them to conclude that their findings provided insights into the mechanisms of astrocyte-to-neuron reprogramming (Kalinin et al., 2021).

### FTIR Microspectroscopy Assessment of VPA-Induced Changes in Chromatin Components

Fourier transform infrared is a sensitive analytical method that permits detection within a sample of vibrational signals that are characteristic of chemical functional groups and that tend to absorb infrared (IR) radiation within a specific wavenumber range (cm^–1^). This methodology is a useful tool for several analytical purposes including those in the biological realm (Singh et al., 2012). Modern technology allows the absorption of an IR light beam that passes through a sample to be examined at all wavenumbers at once, and the use of a Fourier transform algorithm generates a spectral signature in which band peaks are revealed at specific wavenumbers. FTIR spectra for small amounts of solid samples are currently obtained with modern IR microspectroscopes associated with light microscopes.

Regarding the usefulness of FTIR for the analysis of chromatin components, data have been obtained that enabled researchers to discriminate the spectral signatures of DNA of different base compositions, molecular conformations, cytosine methylation abundances and types of histone-DNA complexes (Kelly et al., 2009, 2011; Whelan et al., 2011; Mello and Vidal, 2012; Vidal et al., 2014b; Wood, 2016; Veronezi et al., 2017; Morrish et al., 2019). Investigating how proteins remodel RNA structure by using FTIR monitoring and synchrotron radiation circular dichroism has also been recently proposed (Wien et al., 2021).

**FIGURE 2:**
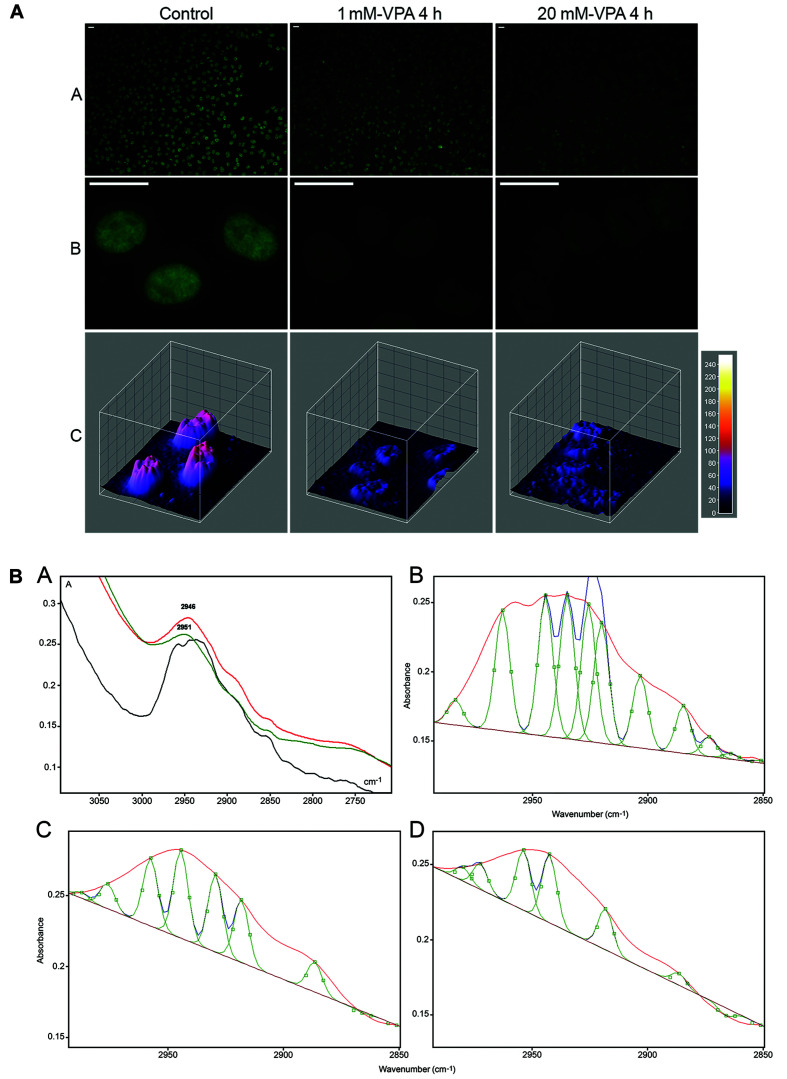
Immunofluorescence signals **(A)** and FTIR spectral window in the 2,990–2,850 cm^–1^ wavenumber range **(B)** for DNA 5mC of HeLa cells cultivated in the presence of VPA. In panel **(A)**, reduction in the DNA 5mC fluorescence signals is shown as assessed visually **(A,B)** and from images obtained with the ImageJ 3D plugin software **(C)**. In panel **(B)**, the area under the band contributed by -CH_3_ signals, which decreases with increasing VPA concentration, is evident in the original normalized spectra **(A)** and after the peak fitting process provided by the Grams/AI 8.0 software [**(C)** (1 mM VPA) and **(D)** (20 mM VPA)] compared to the untreated control **(B)**. In the original spectra shown in panel **(A)**, the curve for the untreated control is represented by the black line, whereas the curves for 1 and 20 mM VPA-treated cells are represented by the red and green lines, respectively [reprinted from Veronezi et al. (2017). PLoS ONE 12(1): e0170740].

In DNA extracted from VPA-treated cells, decreased 5mC abundance, which can lead to changes in chromatin structure (Veronezi et al., 2017; Rocha et al., 2019) was demonstrated using FTIR microspectroscopy. Change in 5mC global abundance was reflected on the infrared spectral window that identifies the symmetrical and anti-symmetrical stretching vibrations of -CH_3_ groups (Veronezi et al., 2017). When VPA elicits a dose-dependent effect on the global abundance of 5mC, as reported and validated immunocytochemically in HeLa cells (Figure 2A), such an event could be demonstrated by analyzing FTIR spectral signatures where the area under the absorption band peak at the ∼2,992–2,850 cm^–1^ spectral window diminishes, thus revealing decreased energy absorption and reduced -CH_3_ abundance (Figure 2B; Veronezi et al., 2017). The differences in the number of peaks within the 2,992 and 2,850 cm^–1^ spectral window when using a peak fitting process (Figure 2B) indicate effects promoted by VPA on the DNA methylation levels that affect the chemical environment at the level of the DNA -CH_3_ stretching vibrations and the chromatin structure (Veronezi et al., 2017).

Currently, studies on FTIR spectral profiles and optical anisotropy imaging obtained from dried mixtures of VPA with DNA, histone H1 and histone H3 have demonstrated a possible interaction of the drug with these chromatin components *in vitro* (Vidal and Mello, 2020). Changes were detected in the textural superstructure of the DNA with a reduction in its molecular order and an effect on its patterns of crystallization. Although the drug does no enter the DNA double helix or bind electrostatically or through hydrogen bonds to DNA, the affinity of VPA to the DNA minor groove was hypothesized to occur through van der Waals interactions. FTIR also provided evidence that VPA can differentially interact with the highly lysine-rich histone H1 and the lysine-poor nucleosome core histone H3, affecting their conformations (Vidal and Mello, 2020). The electrostatic binding of VPA to histone H1, particularly to lysine residues of its terminal tails and to aromatic amino acid residues at its globular domain has been previously proposed using equilibrium dialysis assays, fluorescence emission, and circular dichroism (Sargolzaei et al., 2017). FTIR microspectroscopy confirmed small changes in the conformational state of histone H1 especially in mixtures containing elevated concentrations of VPA (Vidal and Mello, 2020). Decreased absorbances in the amide I and amide II band peaks and in their absorbance ratios in histone H3 in the presence of VPA also indicate changes in the conformational state of this histone induced by the drug (Vidal and Mello, 2020).

Whether VPA has the potential to directly interact with DNA and histones in chromatin, affecting its structure, is currently being investigated in our laboratory and is a matter of pharmacological interest.

## Conclusion

Chromatin architecture is complex and dynamically variable in response to a multitude of effects including those provided by the microenvironment. In this context, the action of drugs such as VPA, which affects epigenetic marks particularly on DNA and histones, and thus induces chromatin remodeling, can be assessed with image analysis and FTIR microspectroscopic analysis. The results obtained with these methods, whether accompanied temporally or validated with further complementary assays, will support a better understanding of the implications of changes in chromatin architecture in association with its functionality.

## Author Contributions

MM conceived, designed, and wrote the original draft of this mini-review.

## Conflict of Interest

The author declares that the research was conducted in the absence of any commercial or financial relationships that could be construed as a potential conflict of interest.

## References

[B1] Barski A. Cuddapah S. Cui K. Roh T. Y. Schones D. Wang Z. (2007). High-resolution profiling of histone methylations in the human genome. *Cell* 129:2007. 10.1016/j.cell.2007.05.009 17512414

[B2] Bassani A. Rocha M. A. Rodrigues V. L. C. C. Santos D. S. Nascimento J. D. da Rosa J. A. (2021). Effects of sodium valproate on the chromatin of *Triatoma infestans* (Klug, 1834) (Hemiptera, Reduviidae) under *in vitro* culture conditions. *Acta Histochem.* 123:151695. 10.1016/j.acthis.2021.151695 33571696

[B3] Beyer S. Zhu J. Mayr D. Kuhn C. Schultze S. Hofmann S. (2017). Histone H3 acetyl K9 and histone H3 tri methyl K4 as prognostic markers for patients with cervical cancer. *Int. J. Mol. Sci.* 18:477. 10.3390/ijms18030477 28241481 PMC5372493

[B4] Booth L. Roberts J. L. Poklepovic A. Kirkwood J. Dent P. (2017). HDAC inhibitors enhance the immunotherapy response of melanoma cells. *Oncotarget* 8 83155–83170. 10.18632/oncotarget.17950 29137331 PMC5669957

[B5] Braiteh F. Soriano A. O. Garcia-Manero G. Hong D. Johnson M. M. De Pádua Silva L. (2008). Phase I study of epigenetic modulation with 5-azacytidine and valproic acid in patients with advanced cancers. *Clin. Cancer Res.* 14 6296–6301. 10.1158/1078-0432.CCR-08-1247 18829512 PMC2582814

[B6] Chateauvieux S. Morceau F. Dicato M. Diederich M. (2010). Molecular and therapeutic potential and toxicity of valproic acid. *J. Biomed. Biotechnol.* 2010:479364. 10.1155/2010/479364 20798865 PMC2926634

[B7] Dejligbjerg M. Grauslund M. Litman T. Collins L. Qian X. Jeffers M. (2008). Differential effects of class I isoform histone deacetylase depletion and enzymatic inhibition by belinostat or valproic acid in HeLa cells. *Mol. Cancer* 7:70. 10.1186/1476-4598-7-70 18789133 PMC2553797

[B8] Detich N. Bovenzi V. Szyf M. (2003). Valproate induces replication-independent active DNA demethylation. *J. Biol. Chem.* 278 27586–27592. 10.1074/jbc.M303740200 12748177

[B9] Dong E. Chen Y. Gavin D. P. Grayson D. R. Guidotti A. (2010). Valproate induces DNA demethylation in nuclear extracts from adult mouse brain. *Epigenetics* 5 730–735. 10.4161/epi.5.8.13053 20716949

[B10] Ellinger J. Kahl P. Mertens C. Rogenhofer S. Hauser S. Hartmann W. (2010). Prognostic relevance of global histone H3 lysine 4 (H3K4) methylation in renal cell carcinoma. *Int. J. Cancer* 127 2360–2366. 10.1002/ijc.25250 20162570

[B11] Eyal S. Yagen B. Sobol E. Altschuler Y. Shmuel M. Bialer M. (2004). The activity of antiepileptic drugs as histone deacetylase inhibitors. *Epilepsia* 45 737–744. 10.1111/j.0013-9580.2004.00104.x 15230695

[B12] Fang E. Zhang H. Jin S. (2014). Epigenetics and cervical cancer: from pathogenesis to therapy. *Tumour Biol.* 35 5083–5093. 10.1007/s13277-014-1737-z 24554414

[B13] Felisbino M. B. Gatti M. S. V. Mello M. L. S. (2014). Changes in chromatin structure in NIH 3T3 cells induced by valproic acid and trichostatin A. *J. Cell. Physiol.* 115 1937–1947. 10.1002/jcb.24865 24913611

[B14] Felisbino M. B. Tamashiro W. M. S. C. Mello M. L. S. (2011). Chromatin remodeling, cell proliferation and cell death in valproic acid-treated HeLa cells. *PLoS One* 6:e29144. 10.1371/journal.pone.0029144 22206001 PMC3242782

[B15] Felisbino M. B. Ziemann M. Khurana I. Okabe J. Al-Hasani K. Maxwell S. (2021). Valproic acid influences the expression of genes implicated with hyperglycaemia-induced complement and coagulation pathways. *Sci. Rep.* 11:2163. 10.1038/s41598-021-81794-4 33495488 PMC7835211

[B16] Fiserova J. Efenberkova M. Sieger T. Maninova M. Uhlifova J. Hozak P. (2017). Chromatin organization at the nuclear periphery as revealed by image analysis of structured illumination microscopy data. *J. Cell Sci.* 130 2066–2077. 10.1242/jcs.198424 28476938

[B17] Fukuchi M. Nii T. Ishimaru N. Minamino A. Hara D. Takasaki I. (2009). Valproic acid induces up- or down-regulation of gene expression responsible for the neuronal excitation and inhibition in rat cortical neurons through its epigenetic actions. *Neurosci. Res.* 65 35–43. 10.1016/j.neures.2009.05.002 19463867

[B18] Ganai S. A. Kalladi S. M. Mahadevan V. (2015). HDAC inhibition through valproic acid modulates the methylation profiles in human embryonic kidney cells. *J. Biomol. Struct. Dynamics* 33 1185–1197. 10.1080/07391102.2014.938247 25012937

[B19] Gialitakis M. Arampatzi P. Makatounakis T. Papamatheakis J. (2010). Gamma interferon-dependent transcriptional memory via relocalization of a gene locus to PML nuclear bodies. *Mol. Cell. Biol.* 30 2046–2056. 10.1128/MCB.00906-09 20123968 PMC2849471

[B20] Göttlicher M. Minucci S. Zhu P. Kramer O. H. Schimpf A. Giavara S. (2001). Valproic acid defines a novel class of HDAC inhibitors inducing differentiation of transformed cells. *EMBO J.* 20 6969–6978. 10.1093/emboj/20.24.6969 11742974 PMC125788

[B21] Gu S. Tian Y. Chlenski A. Salwen H. R. Lu Z. Raj J. U. (2012). Valproic acid shows potent antitumor effect with alteration of DNA methylation in neuroblastoma. *Anticancer Drugs* 23 1054–1066. 10.1097/CAD.0b013e32835739dd 22863973 PMC3710400

[B22] Heers H. Stanislaw J. Harrelson J. Lee M. W. (2018). Valproic acid as an adjunctive therapeutic agent for the treatment of breast cancer. *Eur. J. Pharmacol.* 835 61–74. 10.1016/j.ejphar.2018.07.057 30075223

[B23] Kalinin A. A. Hou X. Ade A. S. Fon G. V. Meixner W. Higgins G. A. (2021). Valproic acid-induced changes of 4D nuclear morphology in astrocyte cells. *bioRχiv, Cold Spring Harbor Laboratory* [preprint]. 10.1101/2020.06.29.178202 PMC8684733 33909457

[B24] Kelly J. G. Martin-Hirsch P. L. Martin F. L. (2009). Discrimination of base differences in oligonucleotides using mid-infrared spectroscopy and multivariate analysis. *Anal. Chem.* 81 5314–5319. 10.1021/ac900546m 19499925

[B25] Kelly J. G. Najand G. M. Martin F. L. (2011). Characterisation of DNA methylation status using spectroscopy (mid-IR) versus Raman with multivariate analysis. *J. Biophoton* 4 345–354. 10.1002/jbio.201000085 21520428

[B26] Kortenhorst M. S. Q. Isharwal S. van Diest P. J. Chowdhury W. H. Marlow C. Carducci M. A. (2009). Valproic acid causes dose- and time-dependent changes in nuclear structure in prostate cancer cells *in vitro* and *in vivo*. *Mol. Cancer Ther.* 8 802–809. 10.1158/1535-7163.MCT-08-1076 19372553 PMC2676893

[B27] Kouzarides T. (2007). Chromatin modifications and their function. *Cell* 128 693–705. 10.1016/j.cell.2007.02.005 17320507

[B28] Light W. H. Brickner J. H. (2013). Nuclear pore proteins regulate chromatin structure and transcription memory by a conserved mechanism. *Nucleus* 4 357–360. 10.4161/nucl.26209 23962805 PMC3899124

[B29] Marchion D. C. Bicaku E. Daud A. I. Sullivan D. M. Munster P. N. (2005). Valproic acid alters chromatin structure by regulation of chromatin modulation proteins. *Cancer Res.* 65 3815–3822. 10.1158/0008-5472.CAN-04-2478 15867379

[B30] Marinova Z. Leng Y. Leeds P. Chuang D. M. (2011). Histone deacetylase inhibition alters histone methylation associated with heat shock protein 70 promoter modifications in astrocytes and neurons. *Neuropharmacology* 60 1109–1115. 10.1016/j.neuropharm.2010.09.022 20888352 PMC3036778

[B31] Meganathan K. Jagtap S. Srinivasan S. P. Wagh V. Hescheler J. Hengstler J. (2015). Neuronal developmental gene and miRNA signatures induced by histone deacetylase inhibitors in human embryonic stem cells. *Cell Death Dis.* 6:e1756. 10.1038/cddic.2015.121 PMC4669700 25950486

[B32] Mello M. L. S. Vidal B. C. (2012). Changes in the microspectroscopic characteristics of DNA caused by cationic elements, different base richness and single-stranded form. *PLoS One* 7:e43169. 10.1371/jornal.pone.0043169 PMC3427352 22937023

[B33] Mello M. L. S. Vidal B. C. (2017). The Feulgen reaction: a brief review and new perspectives. *Acta Histochem.* 119 603–609. 10.1016/j.acthis.2017.07.002 28739089

[B34] Milutinovic S. D’Alessio A. C. Detich N. Szyf M. (2007). Valproate induces widespread epigenetic reprogramming which involves demethylation of specific genes. *Carcinogen* 28 560–571. 10.1093/carcin/bgl167 17012225

[B35] Mohammed T. A. Holen K. D. Jaskula-Sztul R. Mulkerin D. Lubner S. J. Schelman W. R. (2011). A pilot phase II study of valproic acid for treatment of low-grade neuroendocrine carcinoma. *The Oncologist* 16 835–843. 10.1634/theoncologist.2011-0031 21632454 PMC3121900

[B36] Morrish R. B. Hermes M. Metz J. Stone N. Pagliara S. Chahwan R. (2019). Single cell imaging of nuclear architecture changes. *Front. Cell Dev. Biol.* 7:141. 10.3389/fcell.2019.00141 31396512 PMC6668442

[B37] Palsamy P. Bidasee K. R. Shinohara T. (2014). Valproic acid suppresses Nrf2/Keap1 dependent antioxidant protection through induction of endoplasmic reticulum stress and *Keap1* promoter DNA demethylation in human lens epithelial cells. *Exp. Eye Res.* 121 26–34. 10.1016/j.exer.2014.01.021 24525405 PMC4293019

[B38] Petrusevski G. Naumov P. Jovanovski G. Bogoeva-Gaceva G. Ng S. W. (2008). Solid-state forms of sodiium valproate, active componente of the anticonvulsant drug Epilim. *Chem. Med. Chem.* 3 1377–1386. 10.1002/cmdc.200800112 18613204

[B39] Phiel C. J. Zhang F. Huang E. Y. Guenther M. G. Lazar M. A. Klein P. S. (2001). Histone deacetylase is a direct target of valproic acid, a potent anticonvulsant, mood stabilizer, and teratogen. *J. Biol. Chem.* 276 36734–36741. 10.1074/jbc.M101287200 11473107

[B40] Rocha M. A. Mello M. L. S. (2020). Sodium valproate (VPA) modulates the methylation status of histone H3 in HeLa cells. 2020 Virtual Meeting on Epigenetics & Chromatin. Cold Spring Harbor Lab. *Abstracts* 2020:251.

[B41] Rocha M. A. Veronezi G. M. B. Felisbino M. B. Gatti M. S. V. Tamashiro W. M. S. C. Mello M. L. S. (2019). Sodium valproate and 5-aza-2’-deoxycytidine differentially modulate DNA demethylation in G1 phase-arrested and proliferative HeLa cells. *Sci. Rep.* 9:18236. 10.1038/s41598-019-54848-x 31796828 PMC6890691

[B42] Russ B. E. Olshanksy M. Smallwood H. S. Li J. Danton A. E. Prier J. E. (2014). Mapping histone methylation dynamics during virus-specific CD8+T cell differentiation in response to infection. *Immunity* 41 853–865. 10.1016/j.immuni.2014.11.001 25517617 PMC4479393

[B43] Santos-Rosa H. Schneider R. Bannister A. J. Sherriff J. Bernstein B. E. Emre N. C. T. (2002). Active genes are tri-methylated at K4 of histone H3. *Nature* 419 407–411. 10.1038/nature01080 12353038

[B44] Sargolzaei J. Rabbani-Chadegani A. Mollaei H. Deezagi A. (2017). Spectroscopic analysis of the interaction of valproic acid with histone H1 in solution and in chromatin structure. *Int. J. Biol. Macromol.* 99 427–432. 10.1016/j.ijbiomac.2017.02.098 28263810

[B45] Shukla S. Tekwani B. L. (2020). Histone deacetylases inhibitors in neurodegenerative diseases, neuroprotection and neuronal differentiation. *Front. Pharmacol.* 11:537. 10.3389/fphar.2020.00537 32390854 PMC7194116

[B46] Singh B. Gautam R. Kumar S. Kumar B. N. V. Nongthomba U. Nandi D. (2012). Application of vibrational microspectroscopy to biology and medicine. *Curr. Sci.* 102 232–244. 10.1039/c8ra04491k

[B47] Veronezi G. M. B. Felisbino M. B. Gatti M. S. V. Mello M. L. S. Vidal B. C. (2017). DNA methylation changes in valproic acid-treated HeLa cells as assessed by image analysis, immunofluorescence and vibrational microspectroscopy. *PLoS One* 12:e0170740. 10.1371/journal.pone.0170740 28114349 PMC5256918

[B48] Vidal B. C. (1984). Polyploidy and nuclear phenotypes in salivary glands of the rat. *Biol. Cell* 50 137–146. 10.1111/j.1768-322x.1984.tb00260.x 6204703

[B49] Vidal B. C. Felisbino M. B. Mello M. L. S. (2014a). “Image analysis of chromatin remodelling,” in *Functional Analysis of DNA and Chromatin*, eds Stockert J. C. Espada J. Blazquez-Castro A. (New York, NY: Humana Press), 10.1007/978-1-62703-706-8_9

[B50] Vidal B. C. Ghiraldini F. G. Mello M. L. S. (2014b). Changes in liver cell DNA methylation status in diabetic mice affect its FT-IR characteristics. *PLoS One* 9:e102295. 10.1371/journal.pone.0102295 25019512 PMC4096918

[B51] Vidal B. C. Mello M. L. S. (2020). Sodium valproate (VPA) interactions with DNA and histones. *Int. J. Biol. Macromol.* 163 219–231. 10.1016/j.ijbmac.2020.06.265 32619665

[B52] Whelan D. R. Bambery K. R. Heraud P. Tobin M. J. Diem M. McNaughton D. (2011). Monitoring the reversible B to A-like transition of DNA in eukaryotic cells using Fourier transform infrared spectroscopy. *Nucleic Acids Res.* 39 5439–5448. 10.1093/nar/gkr175 21447564 PMC3141270

[B53] Wien F. Geinguenaud F. Grange W. Arluison V. (2021). “SRCD and FTIR spectroscopies to monitor protein-induced nucleic acid remodeling,” in *RNA Remodeling Proteins*, ed. Boudvillain M. (New York, NY: Humana Press). 10.1007/978-1-0716-0935-4_6 33201464

[B54] Wood B. R. (2016). The importance of hydration and DNA conformation in interpreting infrared spectra of cells and tissues. *Chem. Soc. Rev.* 45 1980–1998. 10.1007/978-1-0716-0935-4_6 26403652

[B55] Zhang Y. M. Li M. Meng F. Yu Z. Chen Y. Cui G. (2019). Combination of SB431542, CHIR99021 and PDO325901 has a synergistic effect on abrogating valproic acid-induced epithelial-mesenchymal transition and stemness in HeLa, 5637 and SCC-15 cells. *Oncol. Rep.* 41 3545–3554. 10.3892/or.2019.7088 30942451

